# Prolonged Application of High Fluid Shear to Chondrocytes Recapitulates Gene Expression Profiles Associated with Osteoarthritis

**DOI:** 10.1371/journal.pone.0015174

**Published:** 2010-12-29

**Authors:** Fei Zhu, Pu Wang, Norman H. Lee, Mary B. Goldring, Konstantinos Konstantopoulos

**Affiliations:** 1 Department of Chemical and Biomolecular Engineering, The Johns Hopkins University, Baltimore, Maryland, United States of America; 2 Department of Pharmacology and Physiology, The George Washington University Medical Center, Washington, D.C., United States of America; 3 Hospital for Special Surgery, New York, New York, United States of America; 4 Johns Hopkins Physical Sciences in Oncology Center and Institute for NanoBioTechnology, The Johns Hopkins University, Baltimore, Maryland, United States of America; Ohio State University, United States of America

## Abstract

**Background:**

Excessive mechanical loading of articular cartilage producing hydrostatic stress, tensile strain and fluid flow leads to irreversible cartilage erosion and osteoarthritic (OA) disease. Since application of high fluid shear to chondrocytes recapitulates some of the earmarks of OA, we aimed to screen the gene expression profiles of shear-activated chondrocytes and assess potential similarities with OA chondrocytes.

**Methodology/Principal Findings:**

Using a cDNA microarray technology, we screened the differentially-regulated genes in human T/C-28a2 chondrocytes subjected to high fluid shear (20 dyn/cm^2^) for 48 h and 72 h relative to static controls. Confirmation of the expression patterns of select genes was obtained by qRT-PCR. Using significance analysis of microarrays with a 5% false discovery rate, 71 and 60 non-redundant transcripts were identified to be ≥2-fold up-regulated and ≤0.6-fold down-regulated, respectively, in sheared chondrocytes. Published data sets indicate that 42 of these genes, which are related to extracellular matrix/degradation, cell proliferation/differentiation, inflammation and cell survival/death, are differentially-regulated in OA chondrocytes. In view of the pivotal role of cyclooxygenase-2 (COX-2) in the pathogenesis and/or progression of OA *in vivo* and regulation of shear-induced inflammation and apoptosis *in vitro*, we identified a collection of genes that are either up- or down-regulated by shear-induced COX-2. COX-2 and L-prostaglandin D synthase (L-PGDS) induce reactive oxygen species production, and negatively regulate genes of the histone and cell cycle families, which may play a critical role in chondrocyte death.

**Conclusions/Significance:**

Prolonged application of high fluid shear stress to chondrocytes recapitulates gene expression profiles associated with osteoarthritis. Our data suggest a potential link between exposure of chondrocytes/cartilage to abnormal mechanical loading and the pathogenesis/progression of OA.

## Introduction

Osteoarthritis (OA) is a chronic disease characterized by the degeneration or destruction of the articular cartilage tissue that covers and protects the moving joints. The clinical correlates of OA are joint pain, dysfunction and restricted motion. The etiologies of OA include joint dysplasia, genetic and developmental joint abnormalities, ageing and joint injuries [1]. Indeed, excessive chronic or repetitive mechanical loading of articular cartilage has been reported to play a key role in the development and progression of OA [1]. Chondrocytes represent the sole cellular component of cartilage, and regulate its fate due to their ability to synthesize matrix-degrading enzymes and matrix proteins such as collagens and proteoglycans, which are responsible for the tensile strength and compressive resistance, respectively, of cartilage to mechanical loading. Mechanical loads produce hydrostatic pressure and shear stress which causes tensile strain in some direction [2], [3]. Elegant modeling studies have shown that, in addition to hydrostatic pressure, chondrocytes of the superficial and transitional zones are subjected to high and low fluid flow, respectively, whereas cells of the middle and deep radial zones experience little to no fluid flow [2], [3]. These observations suggest that fluid flow or fluid shear stress is a pathophysiologically relevant mechanical signal in cartilage biology.

Fluid shear modulates intracellular signaling in a time-, magnitude- and phenotype-dependent manner. In the vasculature, high levels of laminar shear are atheroprotective, whereas low shear oscillatory flow tends to be atherogenic. In contrast, numerous *in vitro* studies support the concept that low fluid shear (<10 dyn/cm^2^) is chondroprotective [4], whereas high shear stress (>10 dyn/cm^2^) elicits the release of pro-inflammatory cytokines such as interleukin-6 (IL-6) [5], and mediates matrix degradation [4], [6] and chondrocyte cell death [7], [8], [9], which represent earmarks of OA. Predicted fluid flow and fluid shear stress values *in vivo* are lower than those applied *in vitro* by other investigators and us [4], [5], [6], [7], [8], [9]. We and others have documented that fluid shear affects cell responses in a time- and magnitude-dependent manner. For instance, the reduced antioxidant capacity of chondrocytes was detected after a 24-h exposure to a fluid shear stress level of 40 dyn/cm^2^ [7]. Quantitatively similar results were obtained when chondrocytes were subjected to a lower shear stress level (20 dyn/cm^2^) but for an extended (48 h) shear exposure time [7]. As has appropriately been argued in the literature [3], “*it is the cumulative influence of loading histories throughout life that governs the biology of the tissue*”. It is therefore apparent that detection of chondrocyte responses relevant to OA induced by pathological levels of fluid shear encountered *in vivo* would require extremely long time scales (equivalent to those associated with the onset of OA), which are infeasible and impractical in a laboratory setting. Of note, the inter-dependence between the magnitude and duration of shear for chondrocytes is not known. We, therefore, strategically chose the standard approach employed by toxicologists to evaluate the potential toxicity of lifetime exposure of man to a chemical substance [10]; that is, the investigation of supra-physiological concentrations of the chemical, in our case supra-physiological shear stress levels, for an experimentally feasible time scale.

Since OA is often a consequence of excessive mechanical forces [1] and given that the application of high fluid shear to chondrocytes recapitulates some of the earmarks of OA [4], [6], [7], [8], [9], we aimed to screen the gene expression profiles of shear-activated chondrocytes and assess potential similarities with OA chondrocytes. Using cDNA microarrays, we found that 42 of the 131 differentially regulated genes in sheared chondrocytes have been reported previously in OA chondrocytes, and are related to extracellular matrix (ECM)/matrix degradation, cell growth/differentiation, inflammation and cell survival/death. Consistent with the critical role of cyclooxygenase-2 (COX-2) in the development and/or progression of OA *in vivo* [11] and findings on the regulation of shear-induced reactive oxygen species (ROS) [9] and apoptosis *in vitro* [7], we identified a collection of genes that are regulated by shear-induced COX-2, including genes of the histone and cell cycle families, which may play a critical role in chondrocyte death. Taken together, our data suggest that prolonged application of high fluid shear to human T/C-28a2 chondrocytes recapitulates the earmarks of OA, and illustrate a link between high mechanical forces and the development of OA.

## Results

### Differentially expressed genes in shear-activated human chondrocytes

**Figure 1:**
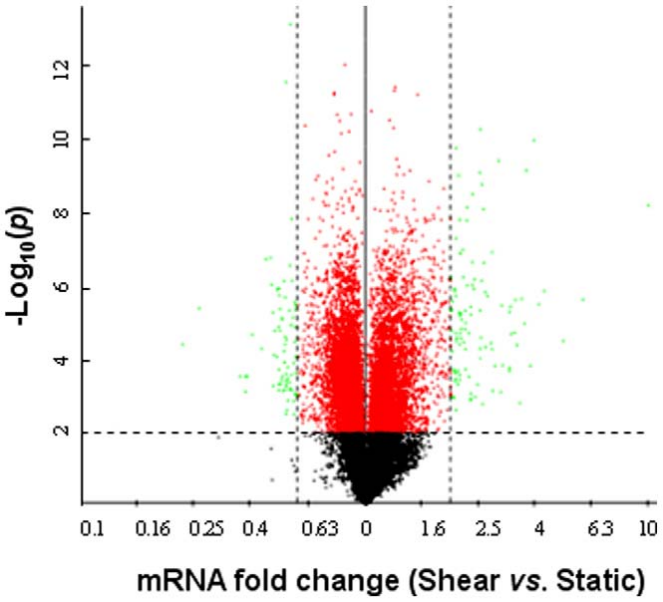
Volcano plot of microarray data. T/C-28a2 chondrocytes were subjected to fluid shear (20 dyn/cm^2^) or static control (0 dyn/cm^2^) conditions for 48 h or 72 h. Three paired samples for each time point were obtained for microarray analysis. The negative log_10_-transformed p-values of the Student's t-test are plotted against the shear to static ratios of fold change in the six-sample experiment. The horizontal bar represents the nominal significant level 0.01 for the Student's t-test (p≤0.01 for 61% of all ESTs represented by the red and green points). The vertical dashed bars denote ≤0.6-fold downregulation (left) or ≥2.0-fold upregulation (right).

**Figure 2:**
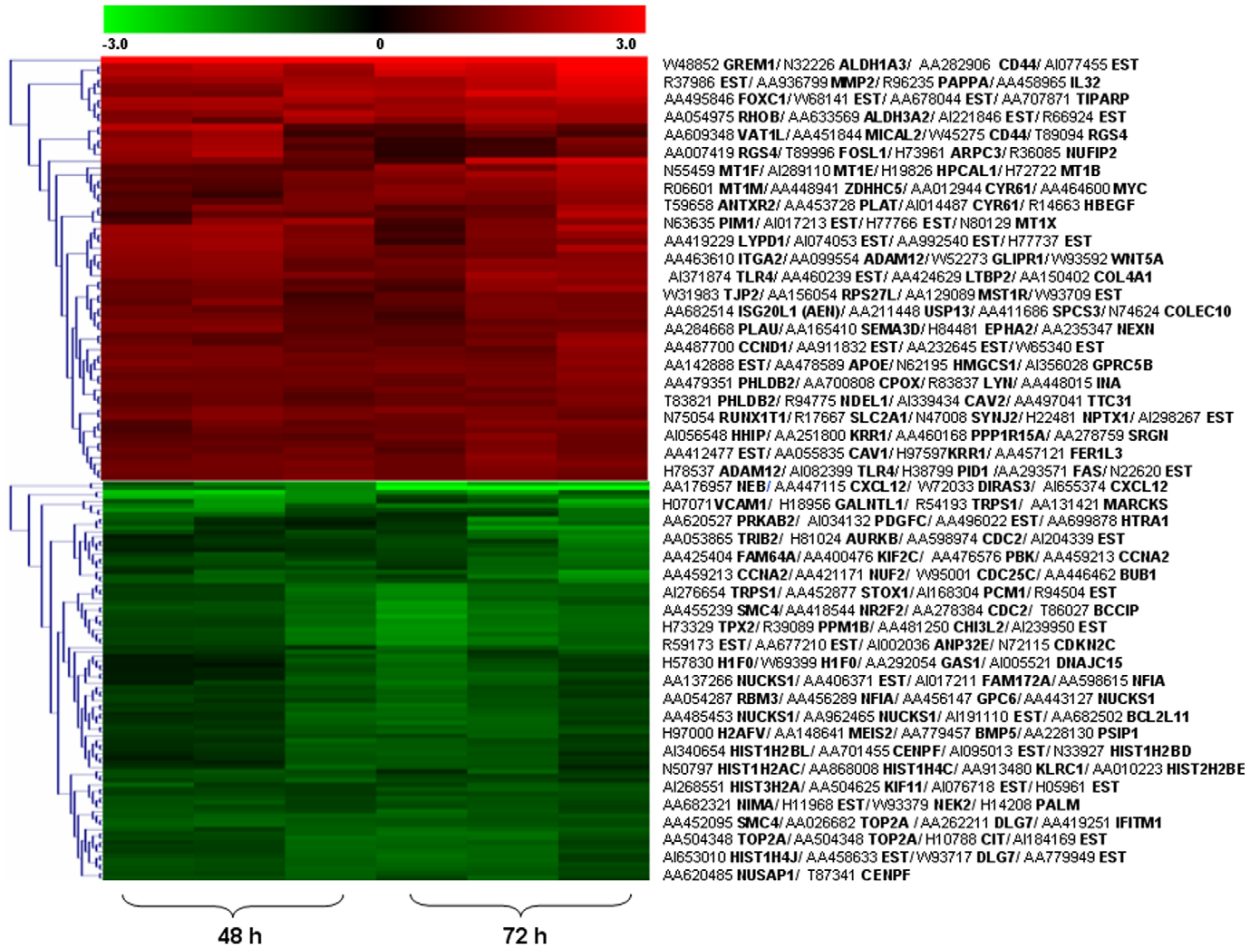
Hierarchical clustering of differentially expressed genes from six sheared and matched static control chondrocyte specimens. Each horizontal row represents a single gene. Up-regulated genes in shear-activated (20 dyn/cm^2^ for 48 h or 72 h) relative to matched static control T/C-28a2 chondrocyte samples are shown in red, whereas down-regulated genes are shown in green.

OA is often a consequence of excessive mechanical loading of cartilage [12], which produces hydrostatic stress, tensile strain and fluid flow [2], [3]. Exposure of human chondrocytes to high fluid shear elicits the release of pro-inflammatory mediators such as interleukin-6 [5], and mediates matrix degradation [4], [6] and apoptosis [7], [8], [9]. In view of accumulating evidence suggesting that prolonged application of high fluid shear recapitulates some of the earmarks of OA, we aimed to identify the differentially-regulated genes in human T/C-28a2 chondrocytes subjected to high fluid shear (20 dyn/cm^2^) versus static (control) conditions (0 dyn/cm^2^) for 48 h and 72 h, using a cDNA microarray technique. In these experiments, total RNA, extracted from control (unsheared) and shear-activated T/C-28a2 cells, was reverse transcribed and labeled with Cy3 and Cy5, respectively, and then hybridized to TIGR 40K human set chips containing 39,936 human expressed sequence tags (ESTs) [7], [9]. As shown in Fig. 1, the expression ratios of 61% of all EST probes between sheared and control genes were statistically significant based on the Student's t-test (p≤0.01). Using SAM with a 5% FDR, 799 probes were found to be differentially regulated between sheared and control specimens. Of these, 98 probes displayed ≥2-fold upregulation, whereas 90 probes showed ≤0.6-fold fold downregulation between sheared and control chondrocytes (Fig. 2, Tables S1 and S2). Of the 98 up-regulated probes, 76, corresponding to 71 non-redundant transcripts, have been sequenced at full-length, whereas the remaining are ESTs (Tables S1). Similarly, of the 90 down-regulated probes, 69, representing 60 non-redundant transcripts, correspond to known genes, whereas the rest are ESTs (Table S2). The differentially-regulated genes with known sequences were classified according to gene ontology (GO), in terms of their involvement in biological processes, and sorted by percentages according to FatiGO (http://www.fatigo.org), a web interface which carries out data mining using GO for DNA microarray data [13], [14] (Fig. S1A and B).

### Comparison of the gene expression profiles between sheared and OA chondrocytes

We next investigated the potential similarities in the gene expression profiles of shear-activated chondrocytes determined in this study and OA chondrocytes reported in the literature. Of the 71 shear-up-regulated genes, 32 have previously been reported to be similarly regulated in OA chondrocytes, accounting for 45% similarity. As shown in Table 1, these genes are related to cell adhesion, cell survival/death, cell growth/differentiation, extracellular matrix (ECM)/matrix degradation, inflammatory response, oxidation/reduction and signal transduction. Although prolonged application of fluid shear increased the mRNA synthesis of TCDD-inducible poly(ADP-ribose) polymerase (PARP-1) in human T/C-28a2 chondrocytes (Table 1), a recent microarray study reported this gene to be down-regulated in OA chondrocytes relative to normal controls [15]. Of note, PARP-1 was found to be up-regulated in rheumatoid arthritis (RA) [16]. Moreover, our data are consistent with prior observations suggesting that RHOB, a member of the Rho GTP-binding protein, is overexpressed in OA [17], [18] and the positive association in the expression levels of RHOB and PARP-1 [19]. Our microarray analysis also identified two additional genes, IL-32 and pappalysin, that are up-regulated in shear-activated chondrocytes as well as in RA [20], [21] but not OA [15]. Of the 60 shear-down-regulated genes, only 3 have been reported to be similarly regulated in OA. A previous microarray study identified two members of the histone family, HIST2H2AA and H3F3B, to be mildly down-regulated in OA knees [22]. Here, we identified 6 new genes of the histone family to be significantly down-regulated in shear-activated chondrocytes (Table 2). Moreover, fluid shear down-regulated the mRNA levels of 9 cell cycle-related genes (Table 2), which may be responsible for chondrocyte apoptosis [9]. Three additional genes, vascular cell adhesion molecule-1 (VCAM-1), chitinase 3-like 2 (CHI3L2) and the chemokine CXCL12 were down-regulated in sheared chondrocytes, although these genes have been reported to be up-regulated in the microarray profiling of OA chondrocytes [15], [22], [23].

**Table 1:** List of differently-regulated genes in shear-activated T/C-28a2 chondrocytes compared to OA chondrocytes obtained from the literature.

*Gene Symbol; (EST)*	*Fold* ±*SD (Shear/Static)*	*References*	*Gene Symbol; (EST)*	*Fold *±*SD (Shear/Static)*	*References*
**Upregulation**			**Upregulation**		
***Cell adhesion***			***Signal transduction***		
CYR61 (AI014487, AA012944)	2.28±0.42	[50], [51]	HBEGF (R14663)	2.08±0.36	[15], [61]
CD44 (W45275 AA282906)	5.14±1.30	[15], [52]	HHIP (AI056548)	2.68±0.72	[62]
***Cell survival/death***			FOSL1 (T89996)	13.15±1.01	[15]
PIM1 (N63635)	2.03±0.40	[15]	EPHA2 (H84481)	2.36±0.21	[15]
MYC (AA464600)	2.15±0.45	[53]	WNT5A (W93592)	2.28±0.05	[15]
FAS (AA293571)	2.53±0.67	[54]	PLAU (AA284668)	2.28±0.15	[63]
*TIPARP (AA707871)*	*3.94*±*0.07*	[*16*] *	LTBP2 (AA424629)	2.36±0.25	[15], [64]
GLIPR1 (W52273)	2.37±0.05	[15]	***Others***		
PPP1R15A (AA460168)	2.57±0.36	[15]	FER1L3 (AA457121)	2.54±0.03	[65]
***Cell growth and differentition***			PLAT (AA453728)	2.19±0.43	[15], [56]
CCND1 (AA487700)	2.37±0.12	[15]	SLC2A1 (R17667)	2.95±0.05	[66]
RHOB (AA054975)	3.70±0.09	[17], [18]	LYN (R83837)	2.18±0.21	[15]
*PAPP-A (R96235)*	*2.94*±*0.71*	[*20*] *	PHLDB2 (AA479351)	2.06±0.13	[15]
GREM1 (W48852)	9.99±0.65	[24]			
***Extracellular matrix and degradation***					
COL4A1 (AA150402)	2.41±0.18	[22]	**Downregulation**		
ADAM12 (AA099554 H78537)	2.52±0.04	[15], [55]	***Signal transduction***		
MMP2 (AA936799)	3.33±1.03	[32]	*CXCL12 (AI655374)*	*0.38±0.09*	[*20]*, [67**]
***Inflammatory Response***			BMP5 (AA779457)	0.57±0.03	[68]
CAV1 (AA055835)	2.64±0.23	[39], [56]	***Others***		
TLR4 (AI371874 AI082399)	2.27±0.06	[15], [57]	*CHI3L2 (AA481250)*	*0.61±0.06*	[*22]*, [32**]
IL32 (AA458965)	2.93±0.83	[21] *	*HTRA1 AA699878*	*0.40±0.10*	[*23*]
***Oxidation/reduction***			MEIS2 (AA148641)	0.56±0.03	[15]
MT1E (AI289110)	2.44±0.68	[58]	*VCAM1 (H07071)*	*0.41±0.07*	[*15*]
MT1X (N80129)	2.11±0.50	[58]	PRKAB2 (AA620527)	0.55±0.10	[15]
APOE (AA478589)	2.14±0.37	[59]			
ALDH1A3 (N32226)	5.91±1.01	[60] #			

35 and 7 genes, up-regulated (≥2-fold) and down-regulated (≤0.6-fold), respectively, in human T/C-28a2 chondrocytes subjected to a shear stress level of 20 dyn/cm^2^ for 48 h and 72 h, were similarly regulated in OA chondrocytes.

*Involved in rheumatoid arthritis.

#murine models.

Genes in italics have reverse regulation in sheared and OA chondrocytes.

**Table 2:** Identification of genes regulated by COX-2 in shear-activated T/C-28a2 chondrocytes.

*Gene Symbol*	*EST*	*Gene ID*	*Shear/static*	*Shear*+*NS398/Shear*
***Cell growth/differentiation***				
PAPP-A	R96235	Pregnancy-associated plasma protein A, pappalysin 1	2.9±0.7	0.4±0.2
***Cell survival/death***				
ISG20L1 (AEN)	AA682514	Apoptosis enhancing nuclease	2.2±0.2	0.4±0.1
FAS [69]	AA293571	Fas (TNF receptor superfamily, member 6)	2.5±0.7	0.3±0.01
***Inflammation***				
CAV1 [36]	AA055835	Caveolin 1	2.6±0.2	0.4±0.01
CAV2	AI339434	Caveolin 2	2.4±0.3	0.5±0.02
***Matrix degradation***				
ADAM12	H78537	ADAM metallopeptidase domain 12	2.6±0.3	0.6±0.03
***Signal transduction***				
EPHA2	H84481	EPH receptor A2	2.4±0.2	0.5±0.01
LTBP2	AA424629	Latent transforming growth factor beta binding protein 2	2.4±0.3	0.5±0.03
***Oxidation/reduction***				
APOE [70]	AA478589	Apolipoprotein E	2.3±0.3	0.5±0.01
***Others***				
ITGA2	AA463610	Integrin, alpha 2	2.2±0.3	0.5±0.03
***Histone Family***				
HIST3H2A	AI268551	Histone cluster 3, H2a	0.5±0.1	1.9±0.02
HIST1H4C	AA868008	Histone cluster 1, H4c	0.5±0.1	1.8±0.02
HIST2H2BE	AA010223	Histone cluster 2, H2be	0.5±0.1	1.9±0.08
HIST1H2BD	N33927	Histone cluster 1, H2bd	0.5±0.1	2.0±0.01
HIST1H4J	AI653010	Histone cluster 1, H4j	0.5±0.1	1.8±0.03
HIST1H2BL	AI340654	Histone cluster 1, H2bl	0.5±0.1	1.7±0.06
***Cell Cycle***				
TPX2	H73329	TPX2, microtubule-associated, homolog	0.6±0.1	1.7±0.2
AURKB	H81024	Aurora kinase B	0.6±0.1	2.1±0.06
NUF2	AA421171	NDC80 kinetochore complex component, homolog	0.6±0.1	1.7±0.03
CDC25C	W95001	Cell division cycle 25 homolog C	0.6±0.1	1.7±0.1
BUB1	AA446462	Budding uninhibited by benzimidazoles 1 homolog	0.6±0.1	1.7±0.04
CDC2	AA278384	Dyclin-dependent kinase 1	0.6±0.07	1.6±0.07
KIF2C	AA400476	Kinesin family member 2C	0.6±0.1	1.7±0.01
NUSAP1	AA620485	Nucleolar and spindle associated protein 1	0.5±0.07	2.0±0.02
CENPF	T87341	Centromere protein F, 350/400 ka (mitosin)	0.5±0.05	1.7±0.04
***Others***				
TOP2A	AA504348	Topoisomerase (DNA) II alpha 170 kDa	0.5±0.04	1.8±0.02
PBK	AA476576	PDZ binding kinase	0.6±0.1	1.7±0.07
STOX1	AA452877	Storkhead box 1	0.6±0.06	1.6±0.3
PALM	H14208	Paralemmin	0.5±0.1	1.8±0.01
CIT	H10788	Citron (rho-interacting, serine/threonine kinase 21)	0.5±0.1	1.8±0.3

Values represent transcript ratios for sheared (20 dyn/cm^2^ for 48 h) to paired static controls (0 dyn/cm^2^ for 48 h) or sheared in the presence of the specific COX-2 inhibitor NS398 (50 µM) to paired sheared controls (20 dyn/cm^2^ for 48 h). Data represent mean ±SD (n≥3).

### Confirmation differential gene expression by qRT-PCR

To validate the expression profiles obtained by microarray analysis, qRT-PCR was used to quantify the mRNA expression levels in sheared and matched static control chondrocytes. We chose to examine the following genes: gremlin in view of consistent literature data suggesting that it is up-regulated in OA chondrocytes [15], [24]; HIST12BD and HIST13H2A, which represent two newly identified genes that are differentially regulated in shear-activated chondrocytes; RHOB in light of conflicting literature data [15]; PAPP-A given their opposite regulation in sheared and OA [15] chondrocytes. qRT-PCR revealed the same gene expression pattern as the microarray analysis in all five genes examined in this work (Table 3).

**Table 3:** Comparison of the transcript ratios of select genes determined by qRT-PCR versus cDNA microarray.

	Shear/Static		Shear+NS398/Shear	
Molecule of interest	qRT-PCR	Microarray	qRT-PCR	Microarray
Gremlin	4.9±0.1	10±0.7	1.4±0.2	ND
HIST12BD	0.5±0.1	0.5±0.1	2.9±0.2	2.0±0.1
HIST13H2A	0.4±0.1	0.5±0.1	1.7±0.1	1.9±0.1
RhoB	7.3±0.1	3.7±0.1	1.0±0.2	0.8±0.1
Pappalysin (PAPP-A)	3.7±0.3	2.9±0.7	0.4±0.2	0.2±0.5

Values represent transcript ratios for sheared (20 dyn/cm^2^ for 48 h) to paired static controls (0 dyn/cm^2^ for 48 h) or sheared in the presence of the specific COX-2 inhibitor NS398 (50 µM) to paired sheared controls (20 dyn/cm^2^ for 48 h). Data represent mean ±SD (n≥3).

### High fluid shear induces IL-1β expression, matrix degradation and reactive oxygen species in human chondrocytes

**Figure 3:**
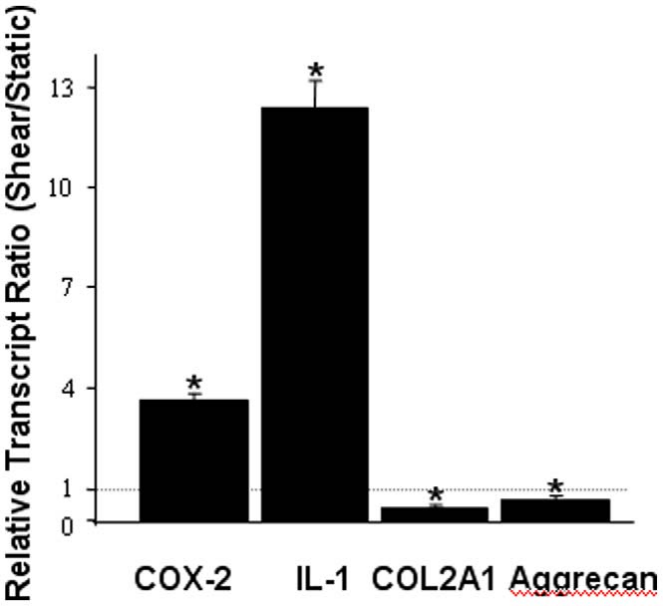
High shear stress induces gene markers of osteoarthritis in human chondrocytes. T/C-28a2 chondrocytes were subjected to fluid shear (20 dyn/cm^2^) or static conditions (0 dyn/cm^2^) for 48 h. qRT-PCR was used to quantify the mRNA transcript ratios of select genes in sheared compared to static control chondrocytes. Data represent the mean±S.D. of n≥3 independent experiments.

Although OA is classified as a non-inflammatory joint disease, prostaglandins and cytokines such IL-1β and IL-6 are believed to play a role in the pathogenesis and progression of disease [11], [25], [26]. In addition to inducing the expression of matrix degrading enzymes, IL-1β also represses the expression of an array of genes associated with the differentiated chondrocyte phenotype, including the type II collagen gene (COL2A1) and aggrecan (*AGC*) [25], [27], [28]. Degradation of aggrecan is considered an important manifestation of OA. We thus evaluated whether prolonged application of high fluid shear to human T/C-28a2 chondrocytes modulates the expression of key marker genes of OA in a manner similar to that detected in OA relative to healthy chondrocytes. As shown in Fig. 3, high fluid shear increases the mRNA levels of COX-2 and IL-1β and concomitantly suppresses those of COL2A1 and AGC in human T/C-28a2 chondrocytes, which is similar to the gene regulation pattern observed in OA chondrocytes [25], [26], [27], [28].

**Figure 4:**
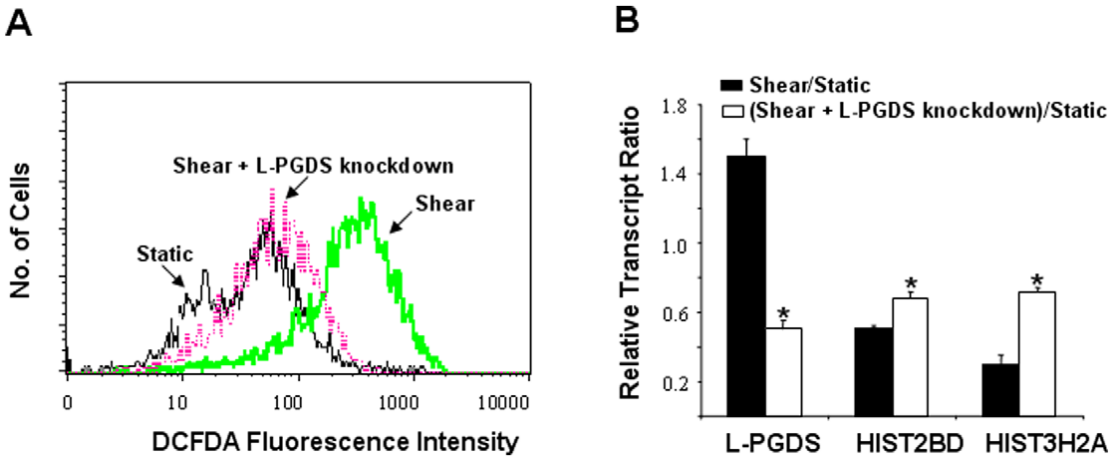
Effects of L-PDGS knockdown on shear-mediated ROS generation and histone regulation in human chondrocytes. T/C-28a2 chondrocytes were subjected to fluid shear (20 dyn/cm^2^) or static conditions (0 dyn/cm^2^) for 48 h. In select experiments, T/C-28a2 cells were transfected with an siRNA oligonucleotide sequence-specific L-PGDS before being subjected to fluid shear. (A) ROS generation was quantified using the DCFDA dye in conjunction with flow cytometry. Histograms are representative of three independent experiments. (B) mRNA transcript ratios for sheared to static control T/C-28a2 chondrocytes (closed bars). In select experiments, the transcript ratio of sheared, L-PGDS knockdown T/C-28a2 cells to static control cells was determined (open bars). Data represent the mean±S.D. of n≥3 independent experiments. *, p<0.05 with respect to shear control.

Accumulating evidence suggests that reactive oxygen species (ROS) contribute to the pathophysiology of OA [29]. ROS generation overwhelms the endogenous antioxidant defense system of chondrocytes, as evidenced by the marked downregulation of a battery of antioxidant genes in OA chondrocytes such as superoxide dismutase, gluthione peroxidase 3 and thioredoxin-interacting protein [22]. Using DCFDA in conjunction with flow cytometry, we determined that high shear stress induces ROS generation in human chondrocytes (Fig. 4A). Knockdown of L-prostaglandin synthase (L-PGDS) via RNA interference abrogated the formation of ROS in sheared T/C-28a2 chondrocytes (Fig. 4). Taken altogether, our data suggest that COX-2-derived PGD_2_ and/or its metabolite 15-deoxy-Δ^12,14^-PGJ_2_ (15d-PGJ_2_) have the ability to generate ROS in sheared T/C-28a2 chondrocytes.

### Genes regulated by COX-2 in shear-activated chondrocytes

**Figure 5:**
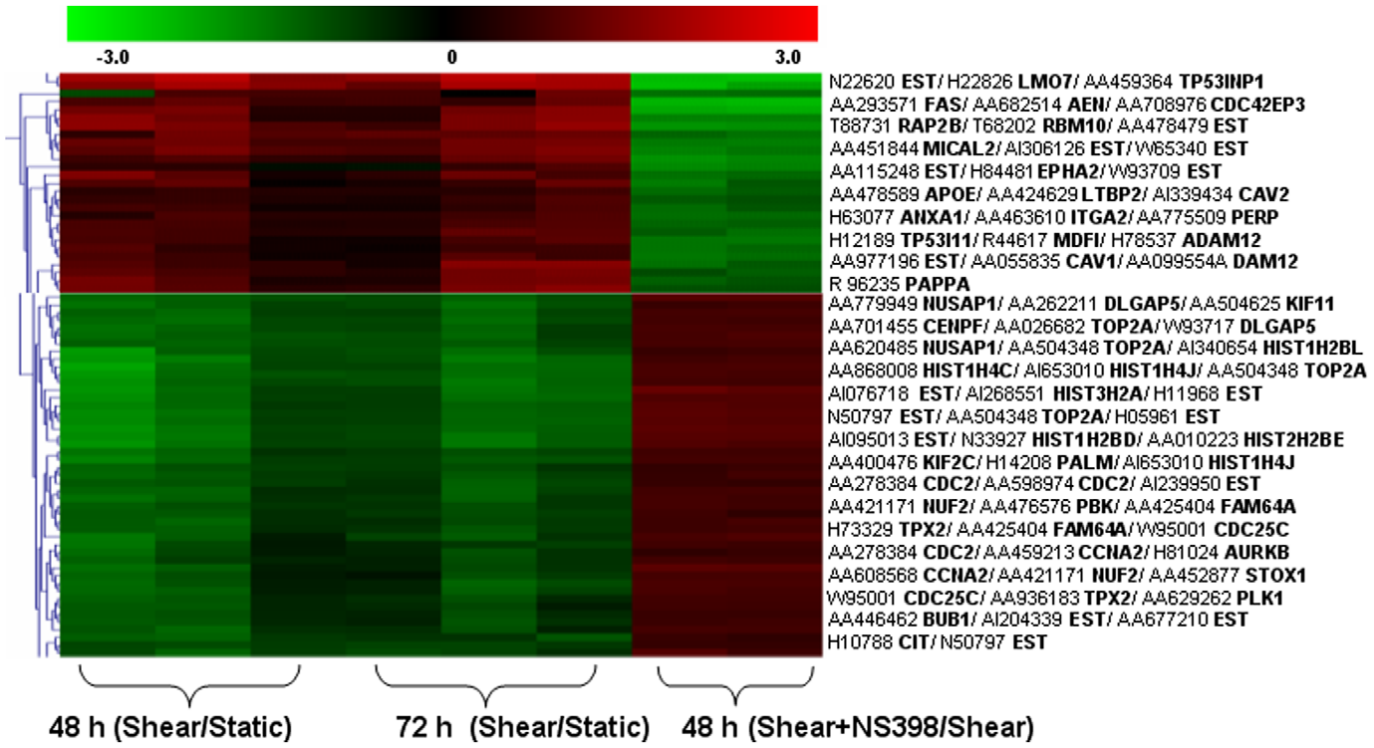
Heat map of genes identified as commonly regulated by COX-2 in shear-activated human T/C-28a2 chondrocytes. Each horizontal row represents a single gene. Up-regulated genes in shear-activated relative to control chondrocyte specimens are shown in red (left upper part). NS398 (50 µM) suppresses the shear-induced COX-2-dependent upregulation of these genes, which are depicted in green (right upper part). Down-regulated genes in sheared relative to static control chondrocytes are shown in green (left lower part). Inhibition of COX-2 activity by NS398 (50 µM) restores the shear-induced COX-2-dependent downregulation of the genes, which are depicted in red (right lower part).

In view of the pivotal role of COX-2 in the regulation of shear-induced inflammation and apoptosis in human chondrocytes [7], [9], we next aimed to identify genes regulated by COX-2 in sheared chondrocytes. The gene transcription profile of T/C-28a2 chondrocytes, subjected to high fluid shear (20 dyn/cm^2^) for 48 h in the presence or absence of the specific COX-2 inhibitor NS398 (50 µM), was determined from microarray experiments using the TIGR MeV software. Our data reveal that the expression pattern of two distinct collections of genes was reversed in sheared chondrocytes incubated with NS398 (Fig. 5). The first collection of genes is positively regulated by COX-2. Thus, inhibition of COX-2 activity by NS398 suppresses the shear-induced, COX-2-dependent upregulation of these genes, which are primarily related to inflammation, matrix degradation and apoptosis (Fig. 5; Table 2; Table S3). The second collection of genes is negatively regulated by COX-2, and as such, inhibition of COX-2 activity restores the shear-induced COX-2-dependent downregulation of these genes back to near basal levels (Fig. 5; Table 2; Table S4). The majority of these genes are histone- and cell cycle- related genes (Table 2; Table S4). To validate the contribution of COX-2 to shear-mediated regulation of histones, T/C-28a2 chondrocytes were transfected, prior to their exposure to high fluid shear, with a siRNA oligonucleotide sequence specific for L-PGDS, which is downstream of COX-2 and responsible for ROS production. This genetic intervention abrogated the shear-mediated downregulation of histone genes (Fig. 4B).

## Discussion

OA is a debilitating disease of the joints characterized by the irreversible erosion of articular cartilage. OA has multiple risk factors including joint dysplasia, genetic and developmental joint abnormalities, ageing and joint injuries [1]. In younger people without genetic/developmental abnormalities, mechanical factors due to trauma are primarily implicated in the initiation and progression of OA lesions [12]. The adult articular chondrocytes, although quiescent in normal cartilage, are able to respond to mechanical forces. Excessive mechanical loading of cartilage producing hydrostatic stress, tensile strain and fluid flow [2], adversely affects chondrocyte function and precipitates OA. The objective of our study was to identify the similarities in the gene expression profiles of shear-activated and OA chondrocytes. Using the cDNA microarray technology, we found that 42 of the 131 differentially regulated genes in sheared chondrocytes have been reported previously in OA chondrocytes, and are related to ECM/matrix degradation, cell growth/differentiation, inflammation and cell survival/apoptosis. It is likely that the 15 histone- and cell cycle- related genes, found to be differentially regulated in sheared chondrocytes, are also involved in OA, since distinct histone [22] and cell cycle [15] related genes were recently reported in microarray studies of OA chondrocytes. In addition, the gene expression patterns of other well-established markers of OA such as COX-2 [11], [30], L-PGDS, IL-1β, COL2A1 and AGC [25], [27], [28], are similar to those detected in sheared chondrocytes. Taken together, at least 60 genes display akin regulation in both sheared and OA chondrocytes.

As shown in Table 1, there were a few genes whose regulation patterns were opposite in shear-activated relative to OA chondrocytes. These differences could be attributed to several reasons such as the distinct etiologies underlying OA, the stage of OA, and the inherent variability of gene expression levels in chondrocytes isolated from different donors. Although high variability might be expected for the disease samples due to different etiology and/or stage of OA, Aigner and coworkers [22] reported a comparable high variability among normal donors. This high variability might also explain why their microarray analysis of OA chondrocytes revealed the downregulation of an array of genes involved in cytokine signaling including IL-1β, IL-8 and leukemia inhibitory factor [22], whereas a recent study showed upregulation of these same genes in OA [15]. Controversy exists among others about whether COL2A1 expression is increased or suppressed in OA cartilage. Aigner and colleagues have suggested that the expression of COL2A1 is suppressed in the upper zones of early OA cartilage, but increased in late-stage OA cartilage relative to normal controls [31], [32]. However, upregulation of collagen genes applies predominantly to those chondrocytes found in the middle and deep zones of OA cartilage, whereas the anabolic phenotype is less obvious in the upper regions [33].

We have demonstrated the critical role of COX-2 in the regulation of shear-induced IL-6 and apoptosis in human chondrocytes [7], [9], [34]. Using cDNA microarrays, we identified genes that were either positively or negatively regulated by COX-2 in shear-activated chondrocytes. The former genes are related to inflammation, matrix degradation and apoptosis. A positive association in the expression levels of COX-2 and caveolin-1 [35], [36] or EPH receptor A2 [37] is supported by findings of other studies employing different cell types. Caveolin-1 and -2 co-localize and form a hetero-oligomeric complex *in vivo* [38]. Moreover, integrin alpha 2 (ITGA2) is associated with caveolin-1 in tumor cells [38]. Interestingly, our data suggest that EPH receptor A2, caveolins-1 and -2 and ITGA2 are under the control of COX-2 in sheared chondrocytes. Caveolin-1 [39] and FAS [39], also positively regulated by COX-2, have been reported to be up-regulated in OA cartilage. In view of our recent observations suggesting that p53 phosphorylation is regulated by COX-2 in sheared chondrocytes [9], it is not surprising that apoptosis enhancing nuclease (AEN) is also under COX-2 control.

Two major classes of genes were identified to be negatively modulated by COX-2 in shear-activated T/C-28a2 chondrocytes: histone and cell-cycle-related genes. We and others have shown that COX-2 overexpression induces cell cycle arrest in diverse cells including chondrocytes, NIH 3T3 fibroblasts, human embryonic kidney 293 cells [9], [40]. Here, we provide evidence for the first time suggesting that overexpression of COX-2 also negatively regulates histone gene expression in sheared chondrocytes. Downregulation of histone gene expression has been detected after DNA damage induced by ionizing radiation in different cells such as human fibroblasts and osteosarcoma [41]. Endogenous degradation of histones was also observed in K562 human leukemic cells after oxidative challenge [42]. The precise role of histones in OA has yet to be defined. Two histone family genes, H2AFO and H3F3B, were shown to be differentially down-regulated in OA chondrocytes relative to healthy control samples, which is in general agreement with our observations in sheared chondrocytes. Moreover, injection of histone H1 into collagen-induced arthritis (CIA) mice dramatically suppressed CIA [43]. Prior work has shown that transcriptional downregulation of histone occurs in parallel with the inhibition of DNA synthesis by p53 [41]. We recently demonstrated that PGD_2_ and/or its metabolite 15d-PGJ_2_ mediate chondrocyte apoptosis via PKA-dependent regulation of p53 phosrphorylation [9]. Indeed, L-PGDS knockdown reverses the shear-mediated histone transcriptional downregulation.

ROS play an important role in the pathogenesis of OA [29]. Excessive levels of ROS generated by abnormal chondrocyte metabolism tip the balance of anabolic and catabolic events, resulting in oxidative stress and loss of homeostasis. We and others have shown that elevated mechanical stress, including shear stress, releases ROS from chondrocytes [9], [12], and that antioxidants repress stress-induced chondrocyte death [7], [12]. L-PGDS knockdown inhibits shear-induced ROS formation, suggesting the involvement of PGD_2_ and/or its metabolite 15d-PGJ_2_ in this process.

In summary, we have demonstrated that prolonged application of high fluid shear to T/C-28a2 chondrocytes recapitulates the earmarks of OA, thereby providing further support to the link between exposure of chondrocytes/cartilage to high mechanical loading and the development of OA. Fluid shear is a well-defined biophysical stimulus for *in vitro* studies of mechanotransduction of articular chondrocytes. Delineating the responses of chondrocytes to high fluid shear may help us understand how OA develops. These studies may also lead to identification of ideal hydrodynamic environments for culturing artificial cartilage in bioreactors.

## Methods

### Reagents

The specific COX-2 inhibitor NS398 was obtained from Cayman Chemical. All other reagents were from Invitrogen, unless otherwise specified.

### Cell Culture and Shear Stress

Human immortalized T/C-28a2 chondrocytes were grown (37°C in 5% CO_2_) on glass slides in 1∶1 Ham's F-12/DMEM medium supplemented with 10% FBS [9], [44]. 24 h prior to the onset of shear stress application, T/C-28a2 cells were incubated in serum-free medium containing 1% Nutridoma-SP (Sigma-Aldrich), a low protein serum replacement that maintains chondrocyte phenotype. T/C-28a2 chondrocytes were subjected to a shear stress level of 20 dyn/cm^2^ for 48 h or 72 h in medium containing 1% Nutridoma-SP by the use of a streamer gold flow device (Flexcell International). In select experiments, the specific COX-2 inhibitor NS398 (50 µM) was added to the medium just before the onset of shear exposure. T/C-28a2 cells have been shown to behave much like primary human chondrocytes when cultured under appropriate conditions [45]. Further evidence suggesting that T/C-28a2 cells represent an appropriate chondrocyte model stems from the significant similarities between human primary chondrocytes and T/C-28a2 cells in the induction of IL-6 synthesis in response to chemical and shear stimulation [34], [46].

### RNA Isolation

Total RNA was isolated using TRIzol, and purified with the RNeasy Mini Kit combined with DNase treatment on a column, according to the manufacturer's protocol (Qiagen).

### Microarray Hybridization

Microarray experiments were performed as previously described [7], [9], [47]. Briefly, total RNA (15 µg), isolated from six independent, paired static and shear-activated T/C-28a2 chondrocyte samples, was reverse transcribed in the presence of random primers and aminoallyl(aa)-dUTP with Superscript II Reverse Transcriptase. The aa-dUTP-labeled cDNAs from sheared and static control samples were coupled to NHS-Cy5 and NHS-Cy3 (GE Healthcare), respectively. Cy5- and Cy-3-labeled targets were mixed, and co-hybridized on the microarray slides printed with a set of 39,936 human ESTs (TIGR 40K Human Set).

### Microarray Data Analysis

Expression levels from individual genes were determined from the scanned microarray slides using TIGR_SpotFinder, and normalized with the total intensity algorithm of the TIGR Microarray Data Analysis System (MIDAS) [47], [48]. Data are presented as mean ± standard deviation (S.D.) using the TIGR Multiexperiment Viewer (MeV). Comparisons between the expression levels of static control and sheared genes were performed using the unpaired Student's t-test, and considered to be statistically significant if p<0.01. Further microarray data analysis involved only statistically significant genes. Differentially expressed genes were then identified using one-class Significance Analysis of Microarray (SAM) at a 5% false discovery rate (FDR) using TIGR MeV [47], [48]. Average linkage hierarchical clustering analysis with a Euclidean distance metric was performed using TIGR MeV [47], [48]. For pathway and functional category classification of the differentially expressed genes, we used the annotations publicly available from the National Center for Biotechnology Information LocusLink database (http://www.ncbi.nlm.nih.gov/LocusLink/), which classifies a gene according to molecular function, biologic process, and cellular component using Gene Ontology categories (http://www.geneontology.org/).

### Quantitative Real-Time PCR (qRT-PCR)

qRT-PCR assays were performed on the iCycler iQ detection system (Biorad) using total RNA, the iScript one-step RT-PCR kit with SYBR green (Biorad) and primers. The GenBank accession numbers and forward (F-) and reverse (R-) primers are as follows:

Gremlin (NM_013372), F-5′-GTATGAGCCGCACAGCCTACA-3′; R-5′-CTCGCTTCAGGTATTTGCGCT-3′

RHOB (NM_004040), F-5′-GGTCCCCTGAGCATGCTTTTCTGA-3′; R-5′-GCCACACTCCCGCGCCAATCTC-3′

PAPP-A (NM_002581), F-5′-CAGAATGCACTGTTACCTGGA-3′; R-5′-GCTGATCCCAATTCTCTTTCA-3′

HIST1H2BD (NM_021063), F-5′-CAAAGAAGGG CTCCAAGAAG-3′; R-5′-TGGTGACGGCCTTGGTGC-3′

HIST3H2A (NM_033445), F-5′-CAGGGTGGCAAGGCGCGCGC-3′; R-5′-TCTTGGGCAGCAGTACGGCC-3′

COX-2 (NM_000963), F-5′-TGAGCATCTACGGTTTGCTG -3′; R-5′-AACTGCTCATCACCCCATTC-3′

Aggrecan (NM_013227), F-5′-ACTTCCGCTGGTCAGATGGA-3′; R-5′-TCTCGTGCCAGATCATCACC-3′

Interleukin-1β (NM_000576), F-5′-ATGGCAGAAGTACCTAAGCTCGC-3′; R-5′-ACACAAATTGCATGGTGAAGTCAGTT-3′

COL2A1 (NM_001844), F-5′-CTGGCTCCCAACACTGCCAACGTC-3′; R-5′-TCCTTTGGGTTTGCAACGGATTGT-3′

L-PGDS (NM_000954), F-5′-GCCTCGCCTCCAACTCGAGC-3′, R-5′-TGCAGCAGCATGGTTCGGGT-3′

GAPDH (NM_002046), F- 5′-CCACCCATGGCAAATTCCATGGCA-3; R-5′- TCTAGACGGCAGGTCAGGTCCACC-3′

GAPDH was used as internal control. Reaction mixtures were incubated at 50°C for 15 min followed by 95°C for 5 min, and then 35 PCR cycles were performed with the following temperature profile: 95°C 15 s, 58°C 30 s, 68°C 1 min, 77°C 20 s. Data were collected at the (77°C 20 s) step to remove possible fluorescent contribution from primer dimers [49].

### Transient Transfection

In RNA interference assays, T/C-28a2 cells were transfected with 100 nM of an siRNA oligonucleotide sequence specific for L-PGDS (SC-41640) or control (SC-44240) siRNA (Santa Cruz). Transfected cells were allowed to recover for at least 12 h in growth medium, and then incubated overnight in serum-free medium containing 1% Nutridoma-SP before their exposure to shear or static conditions.

### ROS Detection

ROS generation was detected by incubating T/C-28a2 chondrocytes with 5-(and-6)-carboxy-2′,7′-dichlorodihydrofluorescein diacetate (carboxy-H2DCFDA; 25 µM in D-PBS containing Ca^2+^/Mg^2+^) for 30 min at 37°C. Cells were next washed with D-PBS lacking Ca^2+^/Mg^2+^, detached from slides by mild trypsinization, re-suspended in D-PBS and examined by flow cytometry.

## Supporting Information

Table S1 **Genes positively regulated by shear stress in human T/C28a2 chondrocytes.**
(PDF)

Table S2 **Genes positively regulated by shear stress in human T/C28a2 chondrocytes.**
(PDF)

Table S3 **Genes positively regulated by COX-2 in human T/C28a2 chondrocytes.**
(PDF)

Table S4 **Genes negatively regulated by COX-2 in human T/C28a2 chondrocytes.**
(PDF)
