# Supplementary material for: Prolonged Application of High Fluid Shear to Chondrocytes Recapitulates Gene Expression Profiles Associated with Osteoarthritis
Source: PLoS One. 2010 Dec 29;5(12):e15174. doi: 10.1371/journal.pone.0015174 (PMC3012157; doi:10.1371/journal.pone.0015174)
Supplement: Table S1 — Genes positively regulated by shear stress in human T/C28a2 chondrocytes. (PDF) [file pone.0015174.s001.pdf]

Supplemental Table S1: Genes positively regulated by shear stress in human T/C28a2 chondrocytes

GOC	EST	Gene symbol	Shear/Static (Folds ± SD)	Description
Cell adhesion and cytosheleton				
	AI014487	CYR61	2.11 ± 0.40	cysteine-rich, angiogenic inducer, 61
	AA012944	CYR61	2.28 ± 0.42	cysteine-rich, angiogenic inducer, 61
	W45275	CD44	3.98 ± 1.29	CD44 molecule (Indian blood group)
	AA282906	CD44	5.14 ± 1.30	CD44 molecule (Indian blood group)
	AA235347	NEXN	2.37 ± 0.16	nexilin (F actin binding protein)
	AA463610	ITGA2	2.24 ± 0.31	integrin, alpha 2
	W31983	TJP2	2.25 ± 0.20	tight junction protein 2 (zona occludens 2)
	H73961	ARPC3	3.32 ± 0.90	actin related protein 2/3 complex
Cell growth and differentition				
	R94775	NDEL1	2.16 ± 0.09	nudE nuclear distribution gene E homolog
	AA487700	CCND1	2.37 ± 0.12	cyclin D1
	AA099554	ADAM12	2.18 ± 0.26	ADAM metallopeptidase domain 12 (meltrin alpha)
	H78537	ADAM12	2.52 ± 0.04	ADAM metallopeptidase domain 12 (meltrin alpha)
	R96235	PAPPA	2.94 ± 0.71	pappalysin-1
Cell survival/death				
	N63635	PIM1	2.03 ± 0.40	pim-1 oncogene
	N75054	RUNX1T1	2.70 ± 0.24	runt-related transcription factor 1; translocated to, 1 (cyclin D-related)
	AA682514	ISG20L1 (AEN)	2.17 ± 0.24	interferon stimulated exonuclease gene 20kDa-like 1
	AA165410	SEMA3D	2.26 ± 0.23	sema domain, immunoglobulin domain (Ig)
	W52273	GLIPR1	2.37 ± 0.05	GLI pathogenesis-related 1 (glioma)
	AA293571	FAS	2.53 ± 0.67	Fas (TNF receptor superfamily, member 6)
	AA460168	PPP1R15A	2.57 ± 0.36	protein phosphatase 1, regulatory (inhibitor) subunit 15A
	AA707871	TIPARP	3.93 ± 0.07	TCDD-inducible poly(ADP-ribose) polymerase
Extracellular matrix and degradation				
	AA936799	MMP2	3.33 ± 1.03	matrix metallopeptidase 2
	AA150402	COL4A1	2.41 ± 0.18	collagen, type IV, alpha 1
Inflammatory				
	AI339434	CAV2	2.21 ± 0.13	caveolin 2
	AI371874	TLR4	2.27 ± 0.06	toll-like receptor 4
	AI082399	TLR4	2.56 ± 0.05	toll-like receptor 4
	AA055835	CAV1	2.64 ± 0.23	caveolin 1
	AA458965	IL32	2.93 ± 0.88	interleukin 32
Oxidation /reduction				

N80129	MT1X	2.10 ± 0.50	metallothionein 1X
N55459	MT1F	2.43 ± 0.69	metallothionein 1F
H72722	MT1B	2.43 ± 0.63	metallothionein I-B
AI289110	MT1E	2.44 ± 0.63	metallothionein 1E
R06601	MT1M	2.54 ± 0.63	metallothionein 1M
AA478589	APOE	2.08 ± 0.02	apolipoprotein E

Signaling Transduction

AA129089	MST1R	2.17 ± 0.25	macrophage stimulating 1 receptor (c-met-related tyrosine kinase)
T59658	ANTXR2	2.18 ± 0.41	anthrax toxin receptor 2
W93592	WNT5A	2.28 ± 0.05	wingless-type MMTV integration site family, member 5A
H84481	EPHA2	2.36 ± 0.21	EPH receptor A2
AA278759	SRGN	2.57 ± 0.48	serglycin
AI056548	HHIP	2.68 ± 0.44	hedgehog interacting protein
AA007419	RGS4	2.73 ± 0.92	regulator of G-protein signaling 4
AA054975	RHOB	3.70 ± 0.09	ras homolog gene family, member B
T89094	RGS4	3.81 ± 1.51	regulator of G-protein signaling 4
W48852	GREM1	9.99 ± 0.65	gremlin 1, cysteine knot superfamily homolog
R14663	HBEGF	2.08 ± 0.36	heparin-binding EGF-like growth factor
AA424629	LTBP2	2.36 ± 0.25	latent transforming growth factor beta binding protein 2
AA464600	MYC	2.15 ± 0.45	v-myc myelocytomatosis viral oncogene homolog (avian)
T89996	FOSL1	3.15 ± 1.01	Fos-like antigen 1
AA495846	FOXC1	4.28 ± 0.54	forkhead box C1

Others

AA479351	PHLDB2	2.06 ± 0.13	pleckstrin homology-like domain, family B
AI356028	GPRC5B	2.07 ± 0.14	G protein-coupled receptor, family C, group 5, member B
AA419229	LYPD1	2.10 ± 0.41	LY6/PLAUR domain containing 1
AA411686	SPCS3	2.14 ± 0.19	signal peptidase complex subunit 3 homolog
AA448015	INA	2.16 ± 0.20	internexin neuronal intermediate filament protein alpha
AA448941	ZDHHC5	2.16 ± 0.62	zinc finger, DHHC-type containing 5
AA211448	USP13	2.16 ± 0.30	ubiquitin specific peptidase 13
AA453728	PLAT	2.19 ± 0.43	plasminogen activator, tissue (tPA) (14)
T83821	PHLDB2	2.20 ± 0.09	pleckstrin homology-like domain, family B member 2
AA156054	RPS27L	2.27 ± 0.36	ribosomal protein S27-like (RPS27L
AA284668	PLAU	2.28 ± 0.15	plasminogen activator, urokinase
N74624	COLEC10	2.32 ± 0.29	collectin sub-family member 10 (C-type lectin)
H19826	HPCAL1	2.52 ± 0.70	hippocalcin-like 1
AA457121	FER1L3	2.54 ± 0.03	fer-1-like 3, myoferlin (C. elegans)
AA251800	KRR1	2.54 ± 0.25	KRR1, small subunit (SSU) processome component,homolog (yeast)

H38799	PID1	2.56 ± 0.18	phosphotyrosine interaction domain containing 1
H97597	KRR1	2.60 ± 0.07	KRR1, small subunit (SSU) processome component,homolog (yeast)
N47008	SYNJ2	2.79 ± 0.15	synaptojanin 2
H22481	NPTX1	2.83 ± 0.50	neuronal pentraxin I
R17667	SLC2A1	2.95 ± 0.05	facilitated glucose transporter
AA497041	TTC31	3.03 ± 0.34	tetratricopeptide repeat domain 31
R36085	NUFIP2	3.13 ± 0.74	nuclear fragile X mental retardation protein interacting protein 2
AA451844	MICAL2	3.24 ± 0.42	microtubule associated monoxygenase, calponin and LIM domain containing 2
AA609348	VAT1L	3.26 ± 0.39	vesicle amine transport protein 1 homolog
AA633569	ALDH3A2	3.57 ± 0.42	aldehyde dehydrogenase 3 family, member A2
N32226	ALDH1A3	5.91 ± 1.01	aldehyde dehydrogenase 1 family, member A3
AA700808	CPOX	2.06 ± 0.12	coproporphyrinogen oxidase
N62195	HMGCS1	2.08 ± 0.07	3-hydroxy-3-methylglutaryl-Coenzyme A synthase 1
R83837	LYN	2.18 ± 0.21	v-yes-1 Yamaguchi sarcoma viral related oncogene homolog

Unknown

AI017213		2.03 ± 0.43	
AI074053		2.07 ± 0.34	
H77766		2.07 ± 0.47	
AA232645		2.10 ± 0.25	
AA142888		2.12 ± 0.03	
W65340		2.14 ± 0.06	
AA992540		2.14 ± 0.37	
W93709		2.17 ± 0.20	
AA911832		2.25 ± 0.26	
H77737		2.36 ± 0.54	
AA460239		2.41 ± 0.16	
AA412477		2.57 ± 0.43	
N22620		2.62 ± 0.15	
AI298267		2.79 ± 0.46	
AI077455		3.30 ± 0.62	
R66924		3.39 ± 0.58	
AI221846		3.41 ± 0.39	
R37986		3.49 ± 0.79	
AA678044		3.68 ± 0.64	
W68141		4.11 ± 0.57	
