# Supplementary material for: Prolonged Application of High Fluid Shear to Chondrocytes Recapitulates Gene Expression Profiles Associated with Osteoarthritis
Source: PLoS One. 2010 Dec 29;5(12):e15174. doi: 10.1371/journal.pone.0015174 (PMC3012157; doi:10.1371/journal.pone.0015174)
Supplement: Table S2 — Genes positively regulated by shear stress in human T/C28a2 chondrocytes. (PDF) [file pone.0015174.s002.pdf]

Supplemental Table S2: Genes negatively regulated by shear stress in human T/C28a2 chondrocytes

GOC	EST	Gene Symbol	Shear/Static (Fold ± SD)	Description
Cell adhesion and cytosheleton				
	AI168304	PCM1	0.60 ± 0.11	pericentriolar material 1
	AA452095	SMC4	0.53 ± 0.08	structural maintenance of chromosomes 4
	AA455239	SMC4	0.56 ± 0.06	structural maintenance of chromosomes 4
	H97000	H2AFV	0.55 ± 0.01	H2A histone family, member V
	AI268551	HIST3H2A	0.54 ± 0.11	histone cluster 3, H2a
	AA131421	MARCKS	0.55 ± 0.12	myristoylated alanine-rich protein kinase C substrate
	N50797	HIST1H2AC	0.53 ± 0.07	histone cluster 1, H2ac
	AA868008	HIST1H4C	0.52 ± 0.08	histone cluster 1, H4c
	AA010223	HIST2H2BE	0.52 ± 0.07	histone cluster 2, H2be
	N33927	HIST1H2BD	0.51 ± 0.07	histone cluster 1, H2bd
	AI653010	HIST1H4J	0.49 ± 0.05	histone cluster 1, H4j
	AI340654	HIST1H2BL	0.49 ± 0.07	histone cluster 1, H2bl
	H57830	H1F0	0.47 ± 0.02	H1 histone family, member 0
	W69399	H1F0	0.45 ± 0.05	H1 histone family, member 0
	AA456147	GPC6	0.57 ± 0.05	glypican 6
	AA176957	NEB	0.24 ± 0.06	nebulin
Cell growth and differentition				
	R39089	PPM1B	0.62 ± 0.06	protein phosphatase 1B (formerly 2C), magnesium-dependent beta isoform
	H73329	TPX2	0.61 ± 0.05	TPX2, microtubule-associated, homolog (Xenopus laevis)
	H81024	AURKB	0.62 ± 0.12	aurora kinase B
	AA421171	NUF2	0.61 ± 0.10	NUF2, NDC80 kinetochore complex component, homolog
	W95001	CDC25C	0.61 ± 0.12	cell division cycle 25 homolog C
	AA446462	BUB1	0.60 ± 0.09	BUB1 budding uninhibited by benzimidazoles 1 homolog
	AA598974	CDC2	0.58 ± 0.06	cyclin-dependent kinase 1
	AA278384	CDC2	0.58 ± 0.07	cell division cycle 2, G1 to S and G2 to M
	AA459213	CCNA2	0.61 ± 0.09	cyclin A2
	AA459213	CCNA2	0.61 ± 0.12	cyclin A2
	AA400476	KIF2C	0.58 ± 0.09	kinesin family member 2C
	AA682321	NEK3	0.56 ± 0.08	NIMA (never in mitosis gene a)-related kinase 3
	W93379	NEK2	0.55 ± 0.06	NIMA (never in mitosis gene a)-related kinase 2
	AA262211	DLG7	0.53 ± 0.05	large homolog 7 (Drosophila)
	AA701455	CENPF	0.51 ± 0.05	centromere protein F
	AA504625	KIF11	0.54 ± 0.12	kinesin family member 11
	W93717	DLG7	0.50 ± 0.03	discs, large homolog 7 (Drosophila)
	AA620485	NUSAP1	0.50 ± 0.07	nucleolar and spindle associated protein 1
	T87341	CENPF	0.49 ± 0.05	centromere protein F
	AA292054	GAS1	0.48 ± 0.03	growth arrest-specific 1

N72115	CDKN2C	0.48 ± 0.02	cyclin-dependent kinase inhibitor 2C

Cell survival/death

T86027	BCCIP	0.61 ± 0.07	BRCA2 and CDKN1A interacting protein
AA148641	MEIS2	0.56 ± 0.03	Meis homeobox 2
AI276654	TRPS1	0.54 ± 0.07	trichorhinophalangeal syndrome I
R54193	TRPS1	0.50 ± 0.06	trichorhinophalangeal syndrome I

Signaling transduction

AA476576	PBK	0.59 ± 0.08	PDZ binding kinase
AA779457	BMP5	0.57 ± 0.03	bone morphogenetic protein 5
H07071	VCAM1	0.41 ± 0.07	vascular cell adhesion molecule 1
AI655374	CXCL12	0.38 ± 0.09	chemokine (C-X-C motif) ligand 12
AA447115	CXCL12	0.26 ± 0.05	chemokine (C-X-C motif) ligand 12
AA418544	NR2F2	0.57 ± 0.04	nuclear receptor subfamily 2, group F, member 2
AA228130	PSIP1	0.56 ± 0.03	PC4 and SFRS1 interacting protein 1
AA456289	NFIA	0.59 ± 0.07	nuclear factor I/A
AA598615	NFIA	0.56 ± 0.05	nuclear factor I/A

Others

AA425404	FAM64A	0.60 ± 0.06	family with sequence similarity 64, member A
AI002036	ANP32E	0.58 ± 0.04	acidic (leucine-rich) nuclear phosphoprotein 32 family, member E
AA962465	NUCKS1	0.57 ± 0.05	similar to PRO2870
AA682502	BCL2L11	0.55 ± 0.06	BCL2-like 11 (apoptosis facilitator)
W72033	DIRAS3	0.40 ± 0.11	GTP-binding RAS-like 3
AA699878	HTRA1	0.40 ± 0.10	HtrA serine peptidase 1
AI034132	PDGFC	0.44 ± 0.06	platelet derived growth factor C
AA452877	STOX1	0.57 ± 0.06	storkhead box 1
H14208	PALM	0.55 ± 0.10	paralemmin
AA053865	TRIB2	0.55 ± 0.10	tribbles homolog 2 (Drosophila)
AA620527	PRKAB2	0.55 ± 0.10	protein kinase, AMP-activated, beta 2 non-catalytic subunit
AA054287	RBM3	0.54 ± 0.07	RNA binding motif (RNP1, RRM) protein 3
AI005521	DNAJC15	0.54 ± 0.08	DnaJ (Hsp40) homolog, subfamily C, member 15
H18956	GALNTL1	0.54 ± 0.07	UDP-N-acetyl-alpha-D-galactosamine: polypeptide N-acetylgalactosaminyltransferase-like 1
AA137266	NUCKS1	0.53 ± 0.03	nuclear casein kinase and cyclin-dependent kinase substrate 1
AA485453	NUCKS1	0.55 ± 0.06	nuclear casein kinase and cyclin-dependent kinase substrate 1
AA443127	NUCKS1	0.53 ± 0.05	nuclear casein kinase and cyclin-dependent kinase substrate 1
H10788	CIT	0.53 ± 0.05	citron (rho-interacting, serine/threonine kinase 21
AA419251	IFITM1	0.52 ± 0.06	interferon induced transmembrane protein 1
AA913480	KLRC1	0.52 ± 0.06	killer cell lectin-like receptor subfamily C

AI017211	FAM172A	0.52 ± 0.04	family with sequence similarity 172, member A
AA481250	CHI3L2	0.61 ± 0.06	chitinase 3-like 2
AA504348	TOP2A	0.51 ± 0.04	topoisomerase (DNA) II alpha 170kDa
AA026682	TOP2A	0.53 ± 0.08	topoisomerase (DNA) II alpha 170kDa
AA504348	TOP2A	0.51 ± 0.07	topoisomerase (DNA) II alpha 170kDa
**Unkown**			
R94504		0.61 ± 0.11	
R59173		0.60 ± 0.09	
AI239950		0.59 ± 0.06	
AA677210		0.58 ± 0.03	
AI204339		0.58 ± 0.07	
AI191110		0.56 ± 0.06	
H11968		0.56 ± 0.09	
AI076718		0.54 ± 0.08	
H05961		0.54 ± 0.06	
AI184169		0.53 ± 0.07	
AA779949		0.51 ± 0.03	
AA406371		0.51 ± 0.05	
AI095013		0.51 ± 0.09	
AA458633		0.49 ± 0.08	
AA496022		0.39 ± 0.09	
