# Supplementary material for: Prolonged Application of High Fluid Shear to Chondrocytes Recapitulates Gene Expression Profiles Associated with Osteoarthritis
Source: PLoS One. 2010 Dec 29;5(12):e15174. doi: 10.1371/journal.pone.0015174 (PMC3012157; doi:10.1371/journal.pone.0015174)
Supplement: Table S3 — Genes positively regulated by COX-2 in human T/C28a2 chondrocytes. (PDF) [file pone.0015174.s003.pdf]

Supplemental Table S3: Genes positively regulated by COX-2 in human T/C28a2 chondrocytes

GOC	EST	Gene Symbol	Shear/Static (Fold ± SD)	Shear+NS398/ Shear (Fold ± SD)	Description
Cell growth and differentition					
	R96235	PAPPA	2.94 ± 0.71	0.35±0.23	
	H78537	ADAM12	2.58 ± 0.25	0.59 ± 0.03	ADAM metallopeptidase domain 12
	AA099554	ADAM12	2.35 ± 0.27	0.57 ± 0.02	ADAM metallopeptidase domain 12
Cell survival/death					
	AA293571	FAS	2.53 ± 0.67	0.31 ± 0.01	Fas (TNF receptor superfamily, member 6)
	AA459364	TP53INP1	2.28 ± 0.79	0.36 ± 0.003	tumor protein p53 inducible nuclear protein 1
	H12189	TP53I11	2.16 ± 0.66	0.53 ± 0.01	tumor protein p53 inducible protein 11
	AA775509	PERP	2.13 ± 0.25	0.56 ± 0.03	TP53 apoptosis effector
	AA682514	AEN	2.07 ± 0.36	0.43 ± 0.03	apoptosis enhancing nuclease
Inflammatory					
	AA055835	CAV1	2.68 ± 0.22	0.40 ± 0.002	caveolin 1
	AI339434	CAV2	2.35 ± 0.25	0.55 ± 0.02	caveolin 2
Oxidation /reduction					
	AA478589	APOE	2.26 ± 0.31	0.55 ± 0.01	apolipoprotein E
Signaling Transduction					
	H84481	EPHA2	2.35 ± 0.39	0.51 ± 0.004	EPH receptor A2
	AA708976	CDC42EP3	2.29 ± 0.42	0.52 ± 0.01	CDC42 effector protein (Rho GTPase binding) 3
	T88731	RAP2B	2.19 ± 0.39	0.37 ± 0.01	RAP2B, member of RAS oncogene family
	AA424629	LTBP2	3.04 ± 0.86	0.52 ± 0.02	latent transforming growth factor beta binding protein 2
Others					
	T68202	RBM10	2.27 ± 0.41	0.37 ± 0.03	RNA binding motif protein 10
	H63077	ANXA1	2.23 ± 0.37	0.54 ± 0.01	annexin A1
	H22826	LMO7	2.19 ± 0.61	0.47 ± 0.01	LIM domain 7
	R44617	MDFI	2.18 ± 0.69	0.26 ± 0.01	MyoD family inhibitor
	AA451844	MICAL2	3.16 ± 0.74	0.60 ± 0.01	microtubule associated monoxygenase
	AA463610	ITGA2	2.50 ± 0.43	0.50 ± 0.01	integrin, alpha 2
Unknown					
	N22620		2.70 ± 0.26	0.56 ± 0.02	
	AI306126		2.53 ± 1.07	0.54 ± 0.02	

W65340	2.37 ± 0.45	0.59 ± 0.01
W93709	2.29 ± 0.34	0.58 ± 0.04
AA115248	2.06 ± 0.41	0.52 ± 0.03
AA478479	2.00 ± 0.75	0.45 ± 0.06
AA977196	2.00 ± 0.62	0.49 ± 0.03
