# Supplementary material for: Prolonged Application of High Fluid Shear to Chondrocytes Recapitulates Gene Expression Profiles Associated with Osteoarthritis
Source: PLoS One. 2010 Dec 29;5(12):e15174. doi: 10.1371/journal.pone.0015174 (PMC3012157; doi:10.1371/journal.pone.0015174)
Supplement: Table S4 — Genes negatively regulated by COX-2 in human T/C28a2 chondrocytes. (PDF) [file pone.0015174.s004.pdf]

Supplemental Table S4: Genes negatively regulated by COX-2 in human T/C28a2 chondrocytes

GOC	EST	Gene Symbol	Shear/Static (Fold ± SD)	Shear+NS398/ Shear (Fold ± SD)	Description
Cell growth and differentition					
	AA936183	TPX2	0.60 ± 0.12	1.70 ± 0.02	microtubule-associated, homolog
	AA446462	BUB1	0.60 ± 0.13	1.74 ± 0.04	budding uninhibited by ben-zimidazoles 1 homolog
	AA608568	CCNA2	0.58 ± 0.16	1.77 ± 0.07	cyclin A2
	W95001	CDC25C	0.58 ± 0.11	1.72 ± 0.10	cell division cycle 25 homolog C
	H73329	TPX2	0.58 ± 0.08	1.72 ± 0.06	microtubule-associated, homolog
	AA629262	PLK1	0.58 ± 0.16	1.82 ± 0.03	polo-like kinase 1
	AA459213	CCNA2	0.57 ± 0.10	1.68 ± 0.04	cyclin A2
	H81024	AURKB	0.56 ± 0.15	1.86 ± 0.09	aurora kinase B
	AA278384	CDC2	0.56 ± 0.08	1.57 ± 0.07	cell division cycle 2
	AA598974	CDC2	0.54 ± 0.09	1.64 ± 0.13	cell division cycle 2
	AA400476	KIF2C	0.54 ± 0.11	1.68 ± 0.004	kinesin family member 2C
	AA504625	KIF11	0.52 ± 0.08	1.76 ± 0.02	kinesin family member 11
	AA620485	NUSAP1	0.50 ± 0.05	1.79 ± 0.01	nucleolar and spindle associated protein 1
	W93717	DLGAP5	0.50 ± 0.08	1.83 ± 0.09	discs, large (Drosophila) homolog-associated protein 5
	AA779949	NUSAP1	0.49 ± 0.05	2.03 ± 0.02	nucleolar and spindle associated protein 1
	AA701455	CENPF	0.49 ± 0.08	1.69 ± 0.04	centromere protein F
	AA262211	DLGAP5	0.48 ± 0.08	1.63 ± 0.05	discs, large (Drosophila) homolog-associated protein 5
Histone					
	AI268551	HIST3H2A	0.46 ± 0.12	1.93 ± 0.02	histone cluster 3, H2a
	N33927	HIST1H2BD	0.45 ± 0.11	2.01 ± 0.01	histone cluster 1, H2bd
	AA010223	HIST2H2BE	0.45 ± 0.12	1.90 ± 0.08	histone cluster 2, H2be
	AA868008	HIST1H4C	0.44 ± 0.12	1.83 ± 0.02	histone cluster 1, H4c
	AI653010	HIST1H4J	0.43 ± 0.09	1.79 ± 0.03	histone cluster 1, H4j
	AI340654	HIST1H2BL	0.43 ± 0.11	1.73 ± 0.06	histone cluster 1, H2bl
Signaling transduction					
	AA476576	PBK	0.54 ± 0.12	1.71 ± 0.07	PDZ binding kinase
Others					
	AA425404	FAM64A	0.57 ± 0.08	1.58 ± 0.14	family with sequence similarity 64, member A
	H10788	CIT	0.57 ± 0.08	1.82 ± 0.25	citron (rho-interacting, serine/ threonine kinase 21)
	AA452877	STOX1	0.55 ± 0.08	1.59 ± 0.34	storkhead box 1
	AA421171	NUF2	0.55 ± 0.12	1.73 ± 0.03	NDC80 kinetochore complex component, homolog
	H14208	PALM	0.50 ± 0.10	1.75 ± 0.01	paralemmin
	AA026682	TOP2A	0.51 ± 0.09	1.77 ± 0.02	topoisomerase (DNA) II alpha

	AA504348	TOP2A	0.46 ± 0.10	1.61 ± 0.04	topoisomerase (DNA) II alpha
**Unknown**					
	AA677210		0.58 ± 0.03	1.54 ± 0.004	
	AI204339		0.56 ± 0.10	1.52 ± 0.08	
	AI239950		0.55 ± 0.08	1.55 ± 0.03	
	H11968		0.49 ± 0.11	1.98 ± 0.08	
	H05961		0.47 ± 0.11	2.00 ± 0.07	
	N50797		0.47 ± 0.11	2.04 ± 0.08	
	AI076718		0.46 ± 0.12	2.22 ± 0.19	
	AI095013		0.46 ± 0.11	2.08 ± 0.04	
